# Impact of glycemic control with sitagliptin on the 2-year progression of arterial stiffness: a sub-analysis of the PROLOGUE study

**DOI:** 10.1186/s12933-016-0472-8

**Published:** 2016-11-03

**Authors:** Hirofumi Tomiyama, Takashi Miwa, Kenshi Kan, Munehide Matsuhisa, Haruo Kamiya, Mamoru Nanasato, Tomoki Kitano, Hiroaki Sano, Jun Ohno, Masato Iida, Masataka Sata, Hirotsugu Yamada, Koji Maemura, Atsushi Tanaka, Toyoaki Murohara, Koichi Node

**Affiliations:** 1 Department of Cardiology, Tokyo Medical University, 6-7-1 Nishi-Shinjuku, Tokyo, Japan; 2 Department of Diabetes, Endocrinology, Metabolism and Rheumatology, Tokyo Medical University, Tokyo, Japan; 3 Division of Diabetes, Metabolism and Endocrinology, Tokyo Medical University Hospital, Tokyo, Japan; 4 Diabetes Therapeutics and Research Center, Tokushima University, Tokushima, Japan; 5 Division of Cardiology, Japanese Red Cross Nagoya Daiichi Hospital, Nagoya, Japan; 6 Cardiovascular Center, Japanese Red Cross Nagoya Daini Hospital, Nagoya, Japan; 7 Department of Cardiology, National Hospital Organization Nagoya Medical Center, Nagoya, Japan; 8 Department of Cardiology, Nagoya Ekisaikai Hospital, Nagoya, Japan; 9 Department of Cardiology, Tsushima Municipal Hospital, Tsushima, Japan; 10 Department of Cardiology, Mitsubishi Nagoya Hospital, Nagoya, Japan; 11 Department of Cardiovascular Medicine, Institute of Biomedical Sciences, Tokushima University Graduate School, Tokushima, Japan; 12 Department of Cardiovascular Medicine, Tokushima University Hospital, Tokushima, Japan; 13 Department of Cardiovascular Medicine, Graduate School of Biomedical Sciences, Nagasaki University, Nagasaki, Japan; 14 Department of Cardiovascular Medicine, Saga University, Saga, Japan; 15 Department of Cardiology, Nagoya University Graduate School of Medicine, Nagoya, Japan

**Keywords:** Arterial stiffness, Dipeptidyl peptidase 4 inhibitor, Glycemic control

## Abstract

**Background:**

No conclusive evidence has been obtained yet on the significance of the effects of dipeptidyl peptidase-4 (DPP-4 inhibitor) treatment on the arterial stiffness in clinical settings. In addition, the effects of good glycemic control on the arterial stiffness have also not been clarified yet. As a sub-analysis of the PROLOGUE study, we examined the effect of a DPP-4 inhibitor (sitagliptin) on the 2-year progression of the arterial stiffness and also to determine the effect of good glycemic control on the rate of progression of the arterial stiffness.

**Methods:**

In the PROLOGUE study, the study participants were either allocated to add-on sitagliptin treatment or to continued treatment with conventional anti-diabetic agents. Among the 463 participants of the PROLOGUE study, we succeeded in measuring the brachial-ankle pulse wave velocity (baPWV) at least two times during the 2-year study period in 96 subjects.

**Results:**

The changes in the baPWV during the study period were similar between the both groups (i.e., with/without staglipitin), overall. On the other hand, when the study subjects were divided into two groups according to the glycemic control status during the study period {good glycemic control group (GC) = hemoglobin (Hb)A1c <7.0 at both 12 and 24 months after the treatment randomization; poor glycemic control group (PC) = HbA1c ≥7.0 at either 12 months, 24 months, or both}, the 2-year increase of the baPWV was marginally significantly larger in the PC group (144 ± 235 cm/s) as compared to that the GC group (−10 ± 282 cm/s) (p = 0.036).

**Conclusion:**

While the present study could not confirm the beneficial effect of sitagliptin per se on the arterial stiffness, the results suggested that good glycemic control appears to be beneficial for delaying the annual progression of the arterial stiffness.

*Trial registration* University Hospital Medical Information Network Clinical Trials Registry UMIN000004490

## Background

Arterial stiffness, as assessed by the pulse wave velocity (PWV), is known as an independent risk factor for cardiovascular disease [1, 2], and PWV is reported as a useful marker to predict the progression of diabetic nephropathy and occurrence of future cardiovascular events in patients with diabetes [3–5]. Some studies have reported that the treatment of diabetes is associated with a reduction of the arterial stiffness [6–9]. Recently, new orally administered dipeptidyl peptidase 4 (DPP-4) inhibitors have become available for the treatment of diabetes [10]. Several experimental studies have demonstrated the beneficial effects of DPP-4 inhibition on diabetic vascular damage [11], however, no conclusive evidence has been obtained yet on the significance of the effects of DPP-4 inhibitor treatment on the arterial stiffness in clinical settings. In addition, while DPP-4 inhibitors are thought to be able to provide better glycemic control without increasing the risk of hypoglycemia [10], the effects of good glycemic control on the arterial stiffness have also not been clarified yet.

The PROLOGUE study was a prospective multicenter study carried out to examine the effect of add-on DPP4 inhibitor therapy on the progression of the carotid intima-media thickness (IMT) over a 2-year follow-up period [12, 13]. In this study, the PWV was also measured in some of the study participants. The arterial stiffness is known to increase annually in subjects [14, 15]. Therefore, we carried out the present study as a sub-analysis of the PROLOGUE study to examine the following

1. The effect of DPP4 inhibitor therapy on the rate of progression of the arterial stiffness, and
2. The effect of good glycemic control on the rate of progression of the arterial stiffness.

## Methods

### Study design and patients

The rationale and design of the PROLOGUE study (University hospital Medical Information Network Center: ID 000004490) have been described previously [12, 13]. In brief, it was a multicenter, prospective, randomized, open-label, blinded-endpoint trial carried out with the participation of 48 Japanese institutions. The trial was approved by the ethics committee of each center and all the patients provided written informed consent. We enrolled 463 patients with T2DM between June 2011 and September 2012. The inclusion criteria and exclusion criteria are described elsewhere [12, 13]. The patients were randomized to either add-on sitagliptin treatment (sitagliptin group) or to continued treatment with conventional anti-diabetic agents (conventional antidiabetic treatment group). In both groups, the target glycemic control level was “maintenance of the HbA1c value at <6.2% or of the fasting blood glucose at <110 mg/dl throughout the study period”. The treatment randomization was conducted based on the age, gender, use of statins, pre-treatment diabetes type (non-pharmacological or pharmacological treatment), HbA1c (<7 or ≥7%), office systolic blood pressure (<135 or ≥135 mmHg) and maximum IMT (<1.0 or ≥1.0 mm) [12, 13]. All the patients were followed up annually for 2 years until September 2014.

### Measurement of the brachial-ankle pulse wave velocity (baPWV)

In the PROLOGUE study, the primary endpoint was the change in the mean common carotid artery (CCA)-IMT at 24 months after treatment randomization. Carotid ultrasound examinations were performed 1 month prior to, or at the time of inclusion of the patient in the study, and then at 12 and 24 months after the treatment randomization. In some of the participating centers, the baPWV was also measured as an ad hoc examination 1 month prior to, or at the time of inclusion of the subject in the study, and then at 12 and 24 months after the treatment randomization (Supplementary text 1 in ref. 13).

The baPWV was measured using a volume-plethysmographic apparatus (Form/ABI; Colin Co. Ltd., Komaki, Japan), as previously described [15, 16]. In brief, occlusion cuffs, connected to both the plethysmographic and oscillometric sensors, were attached to both the upper arms and ankles while the subjects lay in the supine position. The brachial and posterior-tibial arterial pressures were measured using the oscillometric sensors. The measurements were conducted after the subjects had rested for at least 5 min in the supine position, in a temperature-controlled room (24–26 °C) designated exclusively for this purpose. The distance between the sampling points for the brachial-ankle PWV was calculated automatically according to the height of the subject. The path length from the suprasternal notch to the brachium (Lb) was obtained from superficial measurements and was expressed using the equation, Lb = 0.2195 × height of the patient (in centimeters)—2.0734. The path length from the suprasternal notch to the ankle (La) was obtained from superficial measurements and was expressed using the equation, La = (0.8129 × height of the patient (in centimeters) + 12.328). Finally, the following equation was used to calculate the brachial-ankle PWV: brachial-ankle PWV = (La − Lb)/ΔTba. (ΔTba is the time interval between the wavefront of the brachial waveform and that of the ankle waveform).

### Laboratory examinations

Blood samples were taken under the fasting condition from the subjects. The serum malondialdehyde low density-lipoprotein cholesterol (MDA-LDL) concentrations were measured by enzyme-linked immunosorbent assay (SRL Co. Tokyo, Japan). The serum 1,5 anhydroglucitol (1,5 AG) concentrations were measured enzymatically (SRL Co. Tokyo, Japan). The serum C-reactive protein (CRP) concentrations were determined by the latex-aggregation method (SRL Co. Tokyo, Japan), which is a high-sensitivity assay method with a detection threshold of <0.1 mg/L.

### Statistics

Data were expressed as mean ± SD. The delta changes of the variables during the study period were calculated as the values obtained at 12 or 24 months after the treatment randomization minus the values obtained at the baseline. The differences in the measured values between the baseline and at 12 or 24 months after the treatment randomization were assessed by the paired *t* test. McNemar’s non-parametric test was applied for assessment of the differences in the categorical variables between the baseline and at 12 or 24 months after the treatment randomization. The differences between the groups were assessed by the Mann–Whitney U-test or Chi square test. General linear model analysis with post hoc comparison was also applied for assessing the differences in the variables between the groups after adjustments for covariates. The associations among the variables were assessed by univariate linear regression analysis.

All the analyses were conducted using the SPSS software for Windows, version 19.0 J (IBM/SPSS Inc., Chicago, IL); *p* < 0.05 was considered to indicate statistically significant difference.

## Results

Among the 463 subjects who participated in the PROLOGUE study, the baPWV was measured at the baseline in 134 subjects, and again at the end of 1 year and/or 2 years after the treatment randomization in 107 subjects. Among these subjects, the data of 11 subjects with atrial fibrillation and/or ABI < 0.90 were excluded from the study (The accuracy of baPWV is attenuated in these conditions), and finally, the data of 96 subjects were analyzed. In the overall subject population (n = 96), while the HbA1c decreased from 6.9 ± 0.6% (baseline) to 6.7 ± 0.6% (at 24 months after treatment randomization), no significant change of the baPWV (from 1710 ± 318 to 1725 ± 367 cm/s) or blood pressure (from 130 ± 14/72 ± 11 to 131 ± 16/73 ± 10 mmHg) was observed during the study period.

**Fig. 1:**
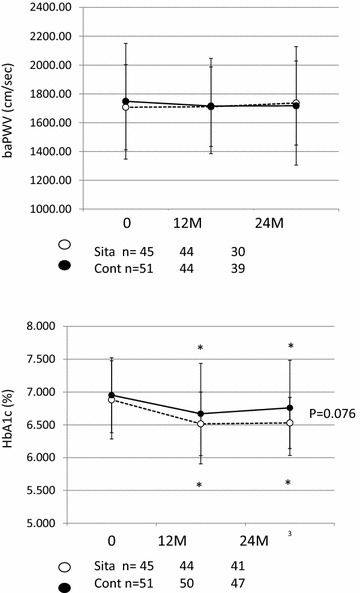
Changes of the brachial-ankle pulse wave velocity and serum hemoglobin A1c levels in subjects assigned to add-on sitagliptin treatment or continuation of therapy with conventional antidiabetic agents. *baPWV* brachial-ankle pulse wave velocity, *HbA1c* hemoglobin A1c, *Sita (empty circles)* subjects assigned to add-on sitagliptin treatment, *Cont (filled circles)* subjects assigned to continued treatment with conventional antidiabetic agents, *0* baseline, *12* *M* 12 months treatment, *24* *M* 24 months treatment, *p < 0.05 vs. baseline

Table 1 shows the clinical characteristics of the study subjects in the sitagliptin and conventional anti-diabetic treatment groups, and no significant differences in the characteristics were observed between the two groups. In both groups, significant decrease of the HbA1c level was observed, and a tendency towards a lower HbA1c value at 24 months after the treatment randomization in the sitagliptin group as compared to that in the conventional anti-diabetic treatment group was observed (p = 0.076) (Fig. 1). On the other hand, no significant change of the baPWV was observed during the study period in either group.

**Table 1:** Clinical characteristics of the study subjects assigned to the add-on sitagliptin group and continued therapy with conventional antidiabetic agents at baseline

Parameters	Sitagliptin (n = 45)	Conventional (n = 51)	p value
Age	70 ± 8	69 ± 9	0.501
Gender (m/f)	29/16	33/18	0.574
BMI	25.5 ± 3.4	25.5 ± 4.4	0.956
SBP (mmHg)	131 ± 12	130 ± 16	0.643
DBP (mmHg)	74 ± 11	71 ± 12	0.256
HR (beats/min)	67 ± 12	69 ± 11	0.389
Current smoker	3	6	0.412
TC (mmol/L)	4.5 ± 0.8	4.5 ± 0.8	0.879
HDL (mmol/L)	1.3 ± 0.3	1.3 ± 0.4	0.996
TG (mmol/L)	1.5 ± 0.7	1.7 ± 1.0	0.176
Crnn (μmol/L)	76 ± 20	75 ± 21	0.805
eGFR (mL/min per 1.73 m^2^)	65 ± 16	67 ± 17	0.675
FBG (mmol/L)	6.8 ± 1.6	7.3 ± 1.9	0.560
Hemoglobin A1c (%)	6.9 ± 0.6	7.0 ± 0.6	0.560
Hypertension	38	40	0.313
Dyslipidemia	32	35	0.484
Cerebrovascular disease	5	7	0.471
Cardiovascular disease	23	33	0.127
Medication for diabetes			
Insulin	0	0	
Biguanides	7	10	0.403
Sulfonylureas	10	11	0.566
Others	21	30	0.162

*n* number of study subjects, *BMI* body mass index, *SBP* systolic blood pressure, *DBP* diastolic blood pressure, *brPP* brachial pulse pressure, *HR* heart rate, *Current smoker* number of current smokers, *TC* serum total cholesterol, *HDL* serum high-density lipoprotein cholesterol, *TG* serum triglyceride, *Crnn* serum creatinine, *eGFR* creatinine-based estimated glomerular filtration rate, *FBG* fasting blood glucose, *Hypertension* number of subjects with hypertension, *Dyslipidemia* number of subjects with dyslipidemia, *Cerebrovascular disease* number of subjects with cerebrovascular disease, *Cardiovascular disease* number of subjects with cardiovascular disease, *Insulin* number of subjects treated with insulin, *Biguanides* number of subjects treated with biguanides, *Sulfonylureas* number of subjects treated with sulfonylureas, *Others* number of subjects treated with antidiabetic agents other than insulin, biguanides or sulfonylureas

**Fig. 2:**
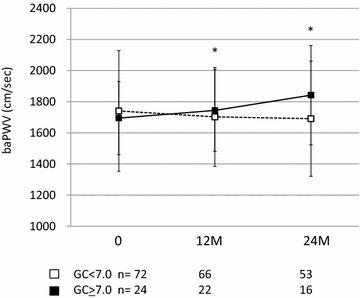
Changes of the brachial-ankle pulse wave velocity in the study subjects with and without good glycemic control during the study period. *GC* *<* *7.0 (empty squares)* subjects with good glycemic control, *GC* *>* *7.0 (filled squares)* subjects with poor glycemic control, *p < 0.05 vs. baseline; other abbreviations are as described in the footnote for Fig. 1

When the study subjects were divided into two groups according to the glycemic control status during the study period {good glycemic control group = hemoglobin (HbA1c) <7.0 at both 12 and 24 months after the treatment randomization; poor glycemic control group = HbA1c ≥7.0 at either 12, 24 months, or both, after the treatment randomization}, while no significant change of the baPWV was noted in the good glycemic control group, the baPWV increased significantly in the poor glycemic control group during the study period (Fig. 2). Table 2 shows the clinical characteristics of the subjects of the good glycemic control and poor glycemic control groups. At the baseline, the subjects of the poor glycemic control group had a tendency toward a higher blood pressure as compared with those of the good glycemic control group. No significant changes of the serum MDA-LDL, hsCRP and 1,5 AG levels were observed in the poor glycemic control group during the study period, while the serum MDA-LDL and 1,5 AG concentrations increased significantly from the baseline to 24 months after the treatment randomization in the good glycemic control group (Table 2).

**Table 2:** Clinical characteristics of the study subjects with and without good glycemic control during the study period at baseline and at the end of 24 months of treatment

Parameters	GC < 7.0 (n = 72)			GC ≥ 7.0 (n = 24)		
	Baseline	24 M	p value	Baseline	24 M	p value
Age	70 ± 9			67 ± 9		
Gender (m/f)	49/23			13/11		
BMI	25.3 ± 3.6	25.4 ± 3.8	ns	26.1 ± 4.9	25.8 ± 5.0	ns
SBP (mmHg)	128 ± 13^#^	130 ± 16	ns	136 ± 16	135 ± 17	ns
DBP (mmHg)	71 ± 10	72 ± 9*	ns	75 ± 13	77 ± 9	ns
HR (beats/min)	68 ± 12	69 ± 13	ns	70 ± 11	70 ± 10	ns
Hemoglobin A1c (%)	6.7 ± 0.4**	6.4 ± 0.3**	p < 0.001	7.5 ± 0.7	7.5 ± 0.7	ns
MDA-LDL (U/L)	106 ± 39	118 ± 34	P = 0.005	111 ± 52	124 ± 42	ns
CRP (ng/mL)	1104 ± 1559	1768 ± 6874	ns	658 ± 745	907 ± 1181	ns
1,5 AG (μg/mL)	17.3 ± 7.2	20.4 ± 8.3	p < 0.001	11.3 ± 7.1	10.4 ± 6.2	ns

*GC* *<* *7.0* subjects with good glycemic control, *GC* *>* *7.0* subjects with poor glycemic control, *24* *M* at the end of 24 months of treatment, *MDA-LDL* serum concentration of malondialdehyde low density-lipoprotein cholesterol concentrations, *CRP* serum concentration of C-reactive protein, *1,5 AG* serum 1,5 anhydroglucitol concentrations, *p value* comparison between the baseline and after 24 months of treatment, ^#^ p = 0.062, * p < 0.050 and ** p < 0.010 vs. subjects without good glycemic control, Other abbreviations are as described in the footnote for Table 1

**Fig. 3:**
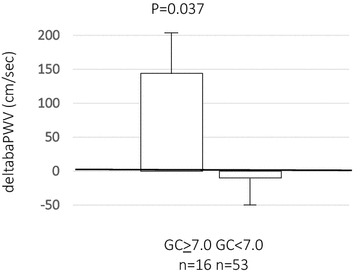
Changes of the brachial-ankle pulse wave velocity from the baseline to 24 months after the treatment randomization in subject groups stratified by the glycemic control status. *deltabaPWV* baPWV value at 24 months after the treatment randomization minus that value at the baseline; other abbreviations are as described in the footnotes for Figs. 1 and 2

We examined the variables that showed a significant relationship to the change of the baPWV from the baseline to the end of the 24-month study period. Among the variables {age, gender, sitagliptin treatment (yes/no) and the clinical characteristics (body mass index, systolic blood pressure, heart rate, serum levels of total cholesterol, high-density lipoprotein cholesterol, triglycerides and HbA1c) at the baseline, at 24 months, and their changes from the baseline to the end of the 24-month study period}, the magnitude of change of the baPWV from the baseline to the end of the 24-month study period was significantly correlated with the magnitudes of change of the systolic blood pressure (R = 0.415, p < 0.001) and heart rate (R = 0.287, p = 0.017) during the same period, and marginally significantly with the HbA1c level at 24 months (R = 0.217, p = 0.075). Then, the magnitude of change of the baPWV from the baseline to 24 months after the treatment randomization (n = 69) was significantly higher in the poor glycemic control group as compared to that in the good glycemic control group (Fig. 3). This difference remained marginally significant even after adjustments for changes of the blood pressure and heart rate from the baseline to the end of the 24-month study period.

The subjects of the good glycemic control group (n = 53) were further subdivided into two groups based on the glycemic control status during the study period {intensive glycemic control group = HbA1c <6.5 at both 12 and 24 months after the treatment randomization; non-intensive, but good glycemic control group = HbA1c >6.5 at either 12, 24 months, or both, after the treatment randomization}. The change of the baPWV from baseline to 24 months after the treatment randomization was similar between the intensive glycemic control (n = 24; 19.5 ± 52.6) and non-intensive, but good glycemic control groups (n = 29; −33.8 ± 56.3) (p = 0.728).

## Discussion

The novel finding of the present study was that the annual rate of progression of arterial stiffness was significantly lower in the subjects with good glycemic control than in those with poor glycemic control.

A recent Cochrane meta-analysis reported that intensive glycemic control was not associated with any significant difference in the all-cause or cardiovascular mortality rates as compared to conventional glycemic control [17]. Therefore, further studies to evaluate the association of the glycemic control status with the risk of cardiovascular events, especially their association with the pathophysiological abnormalities related to cardiovascular risk, are needed. Increased arterial stiffness is thought to act as a risk factor for cardiovascular diseases via inducing increased cardiac afterload, impaired coronary blood flow, microvascular damage, etc. [1, 2, 18]. Thus, it is crucial to examine the effect of the glycemic control status on the arterial stiffness.

Not only a previous cross-sectional study, but also a previous prospective observational study has reported that insufficient glycemic control is related to increased arterial stiffness [19, 20]. On the other hand, controversial results have been reported about the effect of glycemic control on the arterial stiffness [6, 21]. In the overall subject population in this study, the HbA1c value decreased from 6.9 to 6.7%, however, this small reduction of the HbA1c value was not associated with any significant reduction of the arterial stiffness. However, when the subjects were divided into 2 groups based on the glycemic control status during the study period, while the baPWV increased significantly during the 2-year study period in the subjects with poor glycemic control, no significant change was found in the subjects with good glycemic control. Some prospective studies have demonstrated a significant annual progression of arterial stiffness in healthy subjects [14, 15]. This is mentioned in the 3rd paragraph of the Discussion section as follows: While a gender difference in age-related progression of arterial stiffness has been reported [22], in the present study, analysis by the Chi square test demonstrated no significant difference in the gender distribution between the good glycemic control and poor glycemic control groups. In addition, the gender did not affect the change of the baPWV from the baseline to the end of the 24-month study period. Our previous prospective study demonstrated that even mild elevation of the blood sugar levels (>110 mg/dl) accelerated this age-related progression of arterial stiffness [23]. In the present study, sustained good glycemic control status appeared to counteract the age-related progression of arterial stiffness.

In diabetes mellitus, oxidative stress and/or inflammation are thought to contribute to the increase in the arterial stiffness [5, 24]. However, in the present study, the serum CRP, a marker of inflammation, was similar between the subjects with and without good glycemic control. Oddly enough, the serum MDA-LDL, a marker of oxidative stress, was rather higher in the subjects with good glycemic control. Thus, in the present study, good glycemic control appeared to counteract age-related progression of arterial stiffness via some mechanisms other than anti-inflammatory and/or anti-oxidant mechanisms. Apart from these atherogenic factors, advanced end-glycation products (AGEs) have also been reported to contribute to increasing the arterial stiffness in patients with diabetes via accumulating in tissues and the cross-link collagen in arterial wall [5, 24]. In the present study, serum 1,5 AG levels were also higher in the subjects with good glycemic control than in the subjects with poor glycemic control. Hyperglycemia and its duration are thought to contribute to the production of AGEs. Thus, while we did not measure the markers related to AGEs in the present study, one of the plausible mechanisms is that the lower production of AGEs associated with good glycemic control may contribute to delaying the progression of arterial stiffness.

It has not yet been conclusively determined as to which target level of HbA1c, i.e., HbA1c <6.5% or HbA1c <7.0%, might be the more appropriate target level representing good glycemic control [25]. In the present study, when the patients were further subclassified according to the glycemic control status into an intensive glycemic control group (HbA1c <6.5%) and a non-intensive, but good glycemic control group (HbA1c 6.5–7.0%), no significant difference of the changes of the baPWV during the study period was observed between the two groups. While intensive glycemic control is thought to be beneficial against inflammation, oxidative stress, and/or accumulation of AGEs, transient hypoglycemia associated with intensive control might activate the sympathetic tone [5, 25]; thus, intensive glycemic control may have some counteractive effects on the arterial stiffness.

Some studies have reported that pioglitazone improves the vascular functions (i.e., endothelial function and/or arterial stiffness) independent of its effect on glycemia control [8, 9]. It is believed that these beneficial effects may have derived from the anti-inflammatory and/or anti-oxidant effects of pioglitazone. While experimental studies have reported a similar beneficial effect of the DPP4 inhibitors on the vascular functions [11], two single-center studies could not confirm the beneficial effect in clinical settings [26, 27]. Furthermore, no beneficial effect of sitagliptin per se on the arterial stiffness, independent of its effect on the glycemic control, was observed in the present multicenter study either. However, in one reported study by Matsubara et al., a DPP4 inhibitor was shown to improve the endothelial function [28]. Improved endothelial function may affect the arterial stiffness, therefore, a prospective study with a long follow-up duration (more than 2 years) is proposed to clarify whether sitagliptin may exert a beneficial effect on the arterial stiffness, independent of its effect on the glycemic control.

While the TECOS study and EXAMINE study failed to confirm improvement of the cardiovascular outcomes by DPP-4 inhibitor treatment [29], the study period in both of these studies was less than 3 years. Some studies have already demonstrated that DPP-4 inhibitor treatment yields beneficial effects on abnormalities related to cardiovascular disease [30, 31]. Increased arterial stiffness is also an independent risk factor for future cardiovascular events [1, 2, 4]. Thus, further studies with long follow-up periods are needed to examine the effects of DPP-4 inhibitor treatment on the cardiovascular outcomes.

### Study limitations

The present study had some limitations, as follows; (1) The present study was a sub-analysis of PROLOGUE study, and therefore, the CONSORT statement could not be compliant [32]; (2) while the baPWV reflects the stiffness of the middle-to-large arteries, stiffness of the large arteries is thought to be a cardiovascular risk factor. Thus, the beneficial effect of good glycemic control on the progression of large arterial stiffness needs to be examined using the carotid-femoral PWV [1, 4, 18]; (3) The present study was a sub-analysis of the PROLOGUE study, therefore, the number of study subjects (especially the number of subjects with poor glycemic control) was relatively small, and some of them could not obtain data completely. Confirmation of the results of the present study in a larger number of study subjects and also in subjects of other ethnicities is proposed; (4) We could not explore the mechanisms underlying the significant elevation of the serum MDA-LDL concentrations in the subjects with good glycemic control in this study.

## Conclusion

While the present study could not confirm the beneficial effect of sitagliptin per se on the arterial stiffness, good glycemic control (HbA1c <7.0) appears to be beneficial for delaying the annual progression of arterial stiffness. Increased arterial stiffness is thought to contribute to the occurrence of future cardiovascular events via several mechanisms such as increase in cardiac afterload, impaired coronary blood flow, microvascular damage (i.e., pulsatile nephropathy and encephalopathy) and/or atherogenic actions. The next logical step would be to clarify whether the attenuated progression of arterial stiffness associated with sustained good glycemic control might also be associated with a reduced risk of future cardiovascular events.

## Abbreviations

PWV: pulse wave velocity
DPP-4: dipeptidyl peptidase 4
IMT: intima-media thickness
PROLOGUE: Program of Vascular Evaluation under Glucose Control by DPP-4 Inhibitor
T2DM: type 2 diabetes mellitus
baPWV: brachial-ankle pulse wave velocity
CCA: common carotid artery
MDA-LDL: malondialdehude low density-lipoprotein cholesterol
1,5 AG: 1,5 anhydroglucitol
CRP: C-reactive protein

## References

[1] Ben-Shlomo Y, Spears M, Boustred C, May M, Anderson SG, Benjamin EJ, Boutouyrie P, Cameron J, Chen CH, Cruickshank JK, Hwang SJ, Lakatta EG, Laurent S, Maldonado J, Mitchell GF, Najjar SS, Newman AB, Ohishi M, Pannier B, Pereira T, Vasan RS, Shokawa T, Sutton-Tyrell K, Verbeke F, Wang KL, Webb DJ (2014). Aortic pulse wave velocity improves cardiovascular event prediction: an individual participant meta-analysis of prospective observational data from 17,635 subjects. J Am Coll Cardiol.

[2] Vlachopoulos C, Aznaouridis K, Terentes-Printzios D, Ioakeimidis N, Stefanadis C (2012). Prediction of cardiovascular events and all-cause mortality with brachial-ankle elasticity index: a systematic review and meta-analysis. Hypertension.

[3] Theilade S, Lajer M, Jorsal A, Tarnow L, Parving HH, Rossing P (2012). Arterial stiffness and endothelial dysfunction independently and synergistically predict cardiovascular and renal outcome in patients with type 1 diabetes. Diabet Med.

[4] Cruickshank K, Riste L, Anderson SG, Wright JS, Dunn G, Gosling RG (2002). Aortic pulse-wave velocity and its relationship to mortality in diabetes and glucose intolerance: an integrated index of vascular function?. Circulation.

[5] Prenner SB, Chirinos JA (2015). Arterial stiffness in diabetes mellitus. Atherosclerosis..

[6] Bibra Hv, Siegmund T, Ceriello A, Volozhyna M, Schumm-Draeger PM (2009). Optimized postprandial glucose control is associated with improved cardiac/vascular function—comparison of three insulin regimens in well-controlled type 2 diabetes. Horm Metab Res.

[7] Cherney DZ, Perkins BA, Soleymanlou N, Har R, Fagan N, Johansen OE, Woerle HJ, von Eynatten M, Broedl UC (2014). The effect of empagliflozin on arterial stiffness and heart rate variability in subjects with uncomplicated type 1 diabetes mellitus. Cardiovasc Diabetol..

[8] Kiyici S, Ersoy C, Kaderli A, Fazlioglu M, Budak F, Duran C, Gul OO, Sigirli D, Baran I, Tuncel E, Erturk E, Imamoglu S (2009). Effect of rosiglitazone, metformin and medical nutrition treatment on arterial stiffness, serum MMP-9 and MCP-1 levels in drug naive type 2 diabetic patients. Diabetes Res Clin Pract.

[9] Nakamura T, Matsuda T, Kawagoe Y, Ogawa H, Takahashi Y, Sekizuka K, Koide H (2004). Effect of pioglitazone on carotid intima-media thickness and arterial stiffness in type 2 diabetic nephropathy patients. Metabolism..

[10] Green JB, Bethel MA, Armstrong PW, Buse JB, Engel SS, Garg J, Josse R, Kaufman KD, Koglin J, Korn S, Lachin JM, McGuire DK, Pencina MJ, Standl E, Stein PP, Suryawanshi S, Van de Werf F, Peterson ED, Holman RR (2015). TECOS study group. Effect of sitagliptin on cardiovascular outcomes in type 2 diabetes. N Engl J Med.

[11] Avogaro A, de Kreutzenberg S, Fadini G (2014). Dipeptidyl-peptidase 4 inhibition: linking metabolic control to cardiovascular protection. Curr Pharm Des.

[12] Oyama J, Ishizu T, Sato Y, Kodama K, Bando YK, Murohara T, Node K (2014). Rationale and design of a study to evaluate the effects of sitagliptin on atherosclerosis in patients with diabetes mellitus: PROLOGUE study. Int J Cardiol.

[13] Oyama J, Murohara T, Kitakaze M, Ishizu T, Sato Y, Kitagawa Y, Kamiya H, Ajioka M, Ishihara M, Dai K, Nanasato M, Sata M, Maemura K, Tomiyama H, Higashi Y, Kaku K, Yamada H, Matsuhisa M, Yamashita K, Bando YK, Kashihara N, Ueda S, Inoue T, Node K (2016). The effect of sitagliptin on carotid artery atherosclerosis in patients with type 2 diabetes: the PROLOGUE randomized controlled trial. PLoS Med..

[14] Scuteri A, Morrell CH, Orrù M, Strait JB, Tarasov KV, Ferreli LA, Loi F, Pilia MG, Delitala A, Spurgeon H, Najjar SS, AlGhatrif M, Lakatta EG (2014). Longitudinal perspective on the conundrum of central arterial stiffness, blood pressure, and aging. Hypertension.

[15] Tomiyama H, Hashimoto H, Tanaka H, Matsumoto C, Odaira M, Yamada J, Yoshida M, Shiina K, Nagata M, Yamashina A (2010). Continuous smoking and progression of arterial stiffening: a prospective study. J Am Coll Cardiol.

[16] Yamashina A, Tomiyama H, Takeda K, Tsuda H, Arai T, Hirose K, Koji Y, Hori S, Yamamoto Y (2002). Validity, reproducibility, and clinical significance of noninvasive brachial-ankle pulse wave velocity measurement. Hypertens Res.

[17] Hemmingsen B, Lund SS, Gluud C, Vaag A, Almdal TP, Wetterslev J (2013). Targeting intensive glycaemic control versus targeting conventional glycaemic control for type 2 diabetes mellitus. Cochrane Database Syst Rev.

[18] Tomiyama H, Yamashina A (2010). Non-invasive vascular function tests: their pathophysiological background and clinical application. Circ J.

[19] Gunarathne A, Patel JV, Kausar S, Gammon B, Hughes EA, Lip GY (2009). Glycemic status underlies increased arterial stiffness and impaired endothelial function in migrant South Asian stroke survivors compared to European Caucasians: pathophysiological insights from the West Birmingham Stroke Project. Stroke.

[20] Dabelea D, Talton JW, D’Agostino R, Wadwa RP, Urbina EM, Dolan LM, Daniels SR, Marcovina SM, Hamman RF (2013). Cardiovascular risk factors are associated with increased arterial stiffness in youth with type 1 diabetes: the SEARCH CVD study. Diabetes Care.

[21] Yuan C, Lai CW, Chan LW, Chow M, Law HK, Ying M (2014). The effect of diabetes self-management education on body weight, glycemic control, and other metabolic markers in patients with type 2 diabetes mellitus. J Diabetes Res..

[22] Tomiyama H, Yamashina A, Arai T, Hirose K, Koji Y, Chikamori T, Hori S, Yamamoto Y, Doba N, Hinohara S (2003). Influences of age and gender on results of noninvasive brachial-ankle pulse wave velocity measurement—a survey of 12517 subjects. Atherosclerosis..

[23] Tomiyama H, Hashimoto H, Hirayama Y, Yambe M, Yamada J, Koji Y, Shiina K, Yamamoto Y, Yamashina A (2006). Synergistic acceleration of arterial stiffening in the presence of raised blood pressure and raised plasma glucose. Hypertension.

[24] Zieman SJ, Melenovsky V, Kass DA (2005). Mechanisms, pathophysiology, and therapy of arterial stiffness. Arterioscler Thromb Vasc Biol.

[25] Moodahadu LS, Dhall R, Zargar AH, Bangera S, Ramani L, Katipally R (2014). Tight glycemic control and cardiovascular effects in type 2 diabetic patients. Heart Views..

[26] Koren S, Shemesh-Bar L, Tirosh A, Peleg RK, Berman S, Hamad RA, Vinker S, Golik A, Efrati S (2012). The effect of sitagliptin versus glibenclamide on arterial stiffness, blood pressure, lipids, and inflammation in type 2 diabetes mellitus patients. Diabetes Technol Ther..

[27] Zografou I, Sampanis C, Gkaliagkousi E, Iliadis F, Papageorgiou A, Doukelis P, Vogiatzis K, Douma S (2015). Effect of vildagliptin on hsCRP and arterial stiffness in patients with type 2 diabetes mellitus. Hormones (Athens)..

[28] Matsubara J, Sugiyama S, Akiyama E, Iwashita S, Kurokawa H, Ohba K, Maeda H, Fujisue K, Yamamoto E, Kaikita K, Hokimoto S, Jinnouchi H, Ogawa H (2013). Dipeptidyl peptidase-4 inhibitor, sitagliptin, improves endothelial dysfunction in association with its anti-inflammatory effects in patients with coronary artery disease and uncontrolled diabetes. Circ J.

[29] Zannad F, Cannon CP, Cushman WC, Bakris GL, Menon V, Perez AT, Fleck PR, Mehta CR, Kupfer S, Wilson C, Lam H, White WB (2015). EXAMINE Investigators. Heart failure and mortality outcomes in patients with type 2 diabetes taking alogliptin versus placebo in EXAMINE: a multicentre, randomised, double-blind trial. Lancet.

[30] Hibuse T, Maeda N, Kishida K, Kimura T, Minami T, Takeshita E, Hirata A, Nakagawa Y, Kashine S, Oka A, Hayashi M, Nishizawa H, Funahashi T, Shimomura I (2014). A pilot three-month sitagliptin treatment increases serum adiponectin level in Japanese patients with type 2 diabetes mellitus—a randomized controlled trial START-J study. Cardiovasc Diabetol..

[31] Nakamura K, Oe H, Kihara H, Shimada K, Fukuda S, Watanabe K, Takagi T, Yunoki K, Miyoshi T, Hirata K, Yoshikawa J, Ito H (2014). DPP-4 inhibitor and alpha-glucosidase inhibitor equally improve endothelial function in patients with type 2 diabetes: EDGE study. Cardiovasc Diabetol.

[32] Schulz KF, Altman DG, Moher D (2010). Statement: updated guidelines for reporting parallel group randomised trials. Ann Int Med.

